# Non-HDL-cholesterol to HDL-cholesterol ratio is a better predictor of new-onset non-alcoholic fatty liver disease than non-HDL-cholesterol: a cohort study

**DOI:** 10.1186/s12944-018-0848-8

**Published:** 2018-08-21

**Authors:** Kun Wang, Shengshuai Shan, Huabo Zheng, Xiaofang Zhao, Changzhong Chen, Chengyun Liu

**Affiliations:** 1 Department of Geriatrics, Union Hospital, Tongji Medical College, Huazhong University of Science and Technology, Wuhan, 430022 China; 2 Microarray Core Facility, Dana-Farber Cancer Institute, Harvard Medical School, Boston, MA 02215-5450 USA; 3 The First People’s Hospital of Jiangxia District, Wuhan City & Union Jiangnan Hospital, HUST, Wuhan, 430200 China

**Keywords:** nonHDLc/HDLc ratio, Incidence, Non-alcoholic fatty liver disease, Prospective study

## Abstract

**Background:**

The nonHDLc/HDLc ratio (in which nonHDLc is defined as total cholesterol minus HDLc) is positively associated with multiple dyslipidemia-related disorders. This study aimed to determine whether the nonHDLc/HDLc ratio is an independent predictor of new-onset NAFLD (non-alcoholic fatty liver disease) in Chinese population.

**Methods:**

A perspective cohort study consisting of 3374 Chinese adults without liver diseases or metabolic disturbances was performed. Anthropometric parameters and data of metabolic and plasma lipid profile were collected. Univariate and multivariate Cox proportional analyses were carried out to evaluate the association of the nonHDLc/HDLc ratio with incident NAFLD. ROC curve analysis was preformed to compare the predictive value between the nonHDLc/HDLc and the nonHDLc for NAFLD.

**Results:**

Two thousand seven hundred seventeen participants were included in the final analysis. During a median follow-up period of 1.6 years, 264 participants (9.71%) developed NAFLD. After adjustment for potential confounders, a high nonHDLc/HDLc ratio (highest tertile) was associated with elevated risk of NAFLD (HR = 2.66; 95% CI, 1.13–6.24; *P* = 0.025 in female and HR = 2.11; 95% CI, 1.15–3.90; *P* = 0.016 in male). A nonlinear relationship was observed when the nonHDLc/HDLc ratio was ≤3.5. AUC values for nonHDLc/HDLc ratios (0.717 in female and 0.682 in male) were significantly higher than nonHDLc (0.675 in female and 0.653 in male) (*P* = 0.049 in female and *P* = 0.037 in male). In addition, the optimal cut-off value of nonHDLc/HDLc ratio for detection of NAFLD was 2.4 in female and 2.3 in male.

**Conclusions:**

The nonHDLc/HDLc ratio is an independent predictor of NAFLD and a stronger predictor than nonHDLc in Chinese population, which might be expected to better guide early identification of individuals at risk of NAFLD.

## Background

Non-alcoholic fatty liver disease (NAFLD) includes a series of hepatic clinico-pathologic conditions (ranging from hepatic steatosis to steatohepatitis), resembling alcoholic liver disease in individuals without excessive alcohol consumption. With the increasing prevalence of obesity and diabetes, NAFLD has become the most common cause of liver diseases worldwide. And the prevalence rate for NAFLD varies from 25 to 45% in most studies [1–3]. Between 75 million and 100 million individuals in the United States are estimated to have NAFLD and its potential morbidity extends beyond the liver [2]. NAFLD prevalence in most regions of Asia is similar to that in the USA, Australia and New Zealand, and Europe [4]. NAFLD is now considered to be a hepatic manifestation of insulin resistance and a characteristic of metabolic syndrome. Patients with NAFLD overall, and those with non-alcoholic steatohepatitis (NASH), the progressive subtype of NAFLD, in particular, are at increased risk of mortality from liver disease (13%), and more commonly from cardiovascular disease (25%) and malignancy (28%) [2]. Because of the growing burden of this disease, early identification of individuals at high risk for NAFLD helps to prevent the development of NAFLD by primary intervention [5] .

Patients with NAFLD frequently exhibit an atherogenic dyslipidemia that is characterized by hypertriglyceridemia, low levels of high-density lipoprotein cholesterol (HDLc) and low-density lipoprotein cholesterol (LDLc) particles that are smaller and more dense than normal [6–8] . An altered lipid profile may be an important factor for predicting the initiation and progression of NAFLD. However, the lipid abnormalities preceding the new-onset NAFLD were neither tested [9] nor have been fully elucidated [10, 11] . Recently, a longitudinal study, based on a small-scale cohort in the Middle East, suggested that nonHDLc, defined as total cholesterol minus HDLc, is a useful predictor of incident NAFLD [12] .

The nonHDLc to HDLc ratio (nonHDLc/HDLc ratio) could indicate combined lipid problems and was demonstrated as a better predictor for multiple dyslipidemia-related disorders, such as coronary heart disease (CHD) [13], insulin resistance, metabolic syndrome and chronic kidney disease than single lipoprotein or the apoB/apoA1 ratio [14]. A recent observational study of type 2 diabetes also found that the nonHDLc/HDLc ratio had a stronger effect on CHD risk than nonHDLc [15]. However, it is still unclear whether the nonHDLc/HDLc ratio is an equal or a better predictor for the incidence of NAFLD.

In the present study, we aimed to investigate whether the nonHDLc/HDLc can serve as a predictor of incident NAFLD, and, if so, to compare the predictive value for NAFLD between nonHDLc/HDLc and nonHDLc among Chinese.

## Patients and methods

### Study population

All participants were recruited from community-dwelling residents who visited Wuhan Union Hospital for annual medical examinations between February 1, 2014, and May 31, 2014. We excluded participants as follows: (i) subjects with positive serologic markers for hepatitis B, hepatitis C infection, a serum aspartate aminotransferase (AST) to alanine aminotransferase (ALT) ratio > 2, an ALT level more than twice the upper limit of normal, liver cirrhosis, fatty liver disease or malignancy as observed by ultrasonography findings at baseline; (ii) patients who were treated with lipid-lowering agents or who had previous clinical cardiovascular diseases or diabetes; (iii) individuals with a history of alcohol consumption in excess of ≥20 g/day for women and ≥ 30 g/day for men were also excluded to distinguish those with NAFLD. A total of 3374 participants (1731 male and 1643 female) were enrolled in this prospective study.

### Follow-up evaluations

We collected the medical examinations data annually until May 31, 2017 using the same procedures as baseline. Of 3374 participants, 657 did not attend any follow-up assessment, 2717 participants who completed at least one follow-up evaluation were included in the final analysis. Participants who developed NAFLD during follow-up were considered incident NAFLD cases, the follow-up time for incident cases was calculated as the difference between baseline and the examination when incident NAFLD was first identified. For participants who did not develop NAFLD, the follow-up time was calculated as the difference between baseline and the last known follow-up examination.

The collection of medical examination data in Wuhan Union Hospital from February 1, 2014 to May 31, 2017 for this study was approved by the ethics committee of Tongji Medical College, Huazhong University of Science and Technology, and complied with the Declaration of Helsinki of 2008. We verbally informed the participants that the data will be used anonymously for medical study. No informed consent was signed, because the study is observational and the data are anonymized.

### Measurement of variables

Trained investigators obtained lifestyle information, demographic characteristics and previous medical histories through a standard questionnaire. BMI was calculated as weight (kg) divided by height squared (m^2^), and overweight status was defined as a BMI ≥23 kg/m^2^ [16]. Blood pressure was measured with a standard mercury sphygmomanometer with subjects in a seated position after a rest period of at least 5 min, and the average of 3 consecutive readings was used for the analysis. Hypertension was defined as the use of antihypertensive agents, a SBP ≥140 mmHg, or a DBP ≥90 mmHg. Venous blood samples were collected in the morning after an overnight fast and were processed within two hours. An automated chemistry analyser (Beckman Coulter chemistry analyzer AU5800 series, Tokyo, Japan) was used to measure fasting blood glucose, triglycerides (TG), total cholesterol (TC), LDLc, HDLc, and serum AST and ALT levels. HBs Ag, HBs Ab and HCV Ab were measured using a chemiluminescent micro-particle immunoassay and polymerase chain reaction. Abdominal ultrasonography (MINDRAY, DC-8, China) was performed with a 3.5 MHz probe by experienced ultrasonographers blinded to the data of subjects. The main outcome of interest was new-onset NAFLD, which was characterized as increased echogenicity, increased liver-to-kidney contrast, deep beam attenuation, and portal vein blurring [17] .

### Statistical analyses

Summary statistics of the baseline characteristics of all patients and stratification by nonHDLc/HDLc tertiles are expressed as frequencies and proportions for categorical variables and as means and SD or medians and interquartile ranges for continuous variables. The differences between groups were analysed using the Chi-squared test for categorical variables, one-way ANOVA for normally distributed continuous variables, and the Kruskal Wallis test for skewed continuous variables. We examined the relationship between the nonHDLc/HDLc ratio, categorized into tertiles, and the outcomes of NAFLD for the overall population and stratified by gender. Cox proportional hazards models were used to evaluate these relationships both with and without adjustment for confounding variables. The adjusted regression model included baseline age, BMI, SBP, DBP, fasting plasma glucose, TG, ALT, and AST levels and the development of cardiovascular diseases (CVD) or diabetes during follow-up. The TG levels was skewed towards the left, but the results showed no significant change after a Box-Cox transformation for TG. We calculated the hazard ratios (HRs) and 95% confidence intervals (CIs). The lowest tertile was the reference for the nonHDLc/HDLc.

We then applied a generalized additive model to estimate the independent relationship between the nonHDLc/HDLc ratio and the risk of NAFLD, with adjustment for potential confounders. The association was further investigated using a two-piecewise linear model. Then, a likelihood ratio test was conducted to compare the one-line linear model with the two-piecewise linear model to decide which model was suitable for each association. The turning point was determined by a recursive experiment based on the principle of the maximum likelihood method, in other words, using trial and error, including selection of turning points along a pre-defined interval and then choosing the turning point that produced the maximum model likelihood. For convenient clinical use, we designated the nearest half or whole number to be the turning point. Because the nonHDLc/HDLc ratio has no units, we also performed a standard transformation, which indicates how many standard deviations an element is from the mean. To assess the utility of the nonHDLc/HDLc ratio and the nonHDLc value as predictors for NAFLD, we constructed sex-specific receiver operating characteristic (ROC) curves and calculated the areas under the curve (AUC). Bootstrap resampling (Bootstrap resampling times = 10,000) was conducted to calculate 95% CI. We also compared AUC values between different parameters using the Z test. The sensitivity and specificity for predicting NAFLD were calculated by creating dichotomous variables. The Youden index (sensitivity + specificity − 1) and the distance on the ROC curve, calculated as the square root of [(1 – sensitivity)^2^ + (1 − specificity)^2^], were used to determine the appropriate cut-off values. All *P* values were calculated using two-tailed tests of statistical significance with a type I error rate of 5%. Data were analysed with the use of the statistical packages R (The R Foundation; http://www.r-project.org; version 3.4.3) and EmpowerStats (www.empowerstats.com; X&Y Solutions Inc.).

## Results

### Characteristics of individuals by tertiles of the NonHDLc/HDLc ratio

Of 2717 participants included in the final analysis, the mean (standard deviation) age of the cohort was 40.64 (12.00) years, and 51.09% of the participants were male. After a median observation period of 1.61 years, 264 (9.71%) participants developed NAFLD. Table 1 compares the baseline demographic, clinical, and biochemical characteristics of individuals by tertiles of the nonHDLc/HDLc ratio. Significant differences were observed among the groups. Compared with subjects in the lowest tertile of the nonHDLc/HDLc ratio, those in the highest tertile were older, more likely to be male, more obese as assessed by body mass index (BMI), and had higher blood pressures (BPs), higher fasting plasma glucose and worse liver function (higher aspartate aminotransferase (AST) and alanine aminotransferase (ALT) levels). The incidence of NAFLD significantly increased across nonHDLc/HDLc tertiles (3.42% vs. 6.74% vs. 18.98% for tertile 1 vs. tertile 2 vs. tertile 3, respectively).

**Table 1:** Baseline variables and non-alcoholic fatty liver disease (NAFLD) status at the end of follow-up according to the tertiles of nonHDLc/HDL ratio (*n* = 2717)

Variable	nonHDLc/HDL ratio			*P* value
	Tertile 1	Tertile 2	Tertile 3	
	(0.44–1.65; n = 906)	(1.66–2.36; *n* = 905)	(2.37–6.05; *n* = 906)	
Age (yr)	38.83 ± 12.16	40.97 ± 12.19	42.11 ± 11.41	< 0.001
Sex, n (%)				< 0.001
Female	556 (61.37)	458 (50.61)	315 (34.77)	
Male	350 (38.63)	447 (49.39)	591 (65.23)	
Body mass index (kg/m^2^)	21.12 ± 2.36	22.25 ± 2.57	23.47 ± 2.51	< 0.001
Hypertension, n (%)	64 (7.48)	103 (12.06)	142 (16.49)	< 0.001
Systolic BP (mmHg)	109.64 ± 13.33	112.55 ± 13.99	115.80 ± 14.79	< 0.001
Diastolic BP (mmHg)	73.11 ± 8.55	75.09 ± 8.88	77.80 ± 9.59	< 0.001
Fasting plasma glucose (mmol/l)	4.77 ± 0.42	4.88 ± 0.48	4.90 ± 0.51	< 0.001
Triglycerides (mmol/l)	0.83 (0.4)	1.04 (0.59)	1.55 (1)	< 0.001
Total cholesterol (mmol/l)	4.26 ± 0.68	4.67 ± 0.67	5.01 ± 0.79	< 0.001
LDLc (mmol/l)	1.99 ± 0.46	2.49 ± 0.49	2.88 ± 0.66	< 0.001
HDLc (mmol/l)	1.85 ± 0.30	1.56 ± 0.24	1.27 ± 0.22	< 0.001
ALT (U/L)	17.54 ± 9.33	20.19 ± 10.94	23.79 ± 11.81	< 0.001
AST (U/L)	20.49 ± 6.32	20.97 ± 6.41	21.81 ± 6.82	< 0.001
Incident NAFLD, n (%)	31 (3.42)	61 (6.74)	172 (18.98)	< 0.001

Data are expressed as the mean ± SD, median (interquartile range), or percentage

*HDLc* High-density lipoprotein cholesterol.; *BP* Blood pressure; *LDLc* Low-density lipoprotein cholesterol; *ALT* Alanine aminotransferase; *AST* Aspartate aminotransferase

Hypertension was defined as blood pressure ≥ 140/90 mmHg or use of anti-hypertensive treatment

### Unadjusted association between baseline variables and the risk of NAFLD

Table 2 shows the univariate Cox proportional hazards models. The univariate analysis indicated that male sex (HR = 2.70, 95% CI, 2.06–3.54, *P* < 0.001), BMI (HR = 1.34, 95% CI, 1.29–1.39, *P* < 0.001), systolic BP (SBP) (HR = 1.02, 95% CI, 1.01–1.03, *P* < 0.001), diastolic BP (DBP) (HR = 1.04, 95% CI, 1.03–1.06, *P* < 0.001), fasting plasma glucose (HR = 1.40, 95% CI, 1.10–1.78, *P* = 0.005), TG (HR = 1.68, 95% CI, 1.54–1.84, *P* < 0.001), TC (HR = 1.49, 95% CI, 1.29–1.72, *P* < 0.001), LDLc (HR = 1.76, 95% CI, 1.49–2.08, *P* < 0.001), nonHDLc (HR = 1.96, 95% CI, 1.71–2.24, *P* < 0.001), nonHDLc/HDLc (HR = 2.19, 95% CI, 1.94–2.47, *P* < 0.001), ALT (HR = 1.04, 95% CI, 1.04–1.05, *P* < 0.001) and AST (HR 1.04, 95% CI, 1.03–1.05, *P* < 0.001) values were positively correlated with the risk of NAFLD. The HDLc value (HR = 0.15, 95% CI, 0.10–0.22, *P* < 0.001) was negatively correlated with the risk of NAFLD.

**Table 2:** The unadjusted association between baseline variables and NAFLD status at the end of follow-up (*n* = 2717)

Variable	Statistics	Hazard ratio (95% CI)	*P* value
Age (yr)	40.64 ± 12.00	1.01 (1.00, 1.02)	0.049
Sex, n (%)			< 0.001
Female	1329 (48.91)	1.0	
male	1388 (51.09)	2.70 (2.06, 3.54)	
Body mass index (kg/m^2^)	22.28 ± 2.66	1.34 (1.29, 1.39)	< 0.001
Hypertension, n (%)	309 (12.02)	1.58 (1.15, 2.16)	0.004
Systolic BP (mmHg)	112.67 ± 14.27	1.02 (1.01, 1.03)	< 0.001
Diastolic BP (mmHg)	75.34 ± 9.22	1.04 (1.03, 1.06)	< 0.001
Fasting plasma glucose (mmol/l)	4.85 ± 0.48	1.40 (1.10, 1.78)	0.005
Triglycerides (mmol/l)	0.99 (0.48)	1.68 (1.54, 1.84)	< 0.001
Total cholesterol (mmol/l)	4.65 ± 0.78	1.49 (1.29, 1.72)	< 0.001
LDLc (mmol/l)	2.45 ± 0.65	1.76 (1.49, 2.08)	< 0.001
HDLc (mmol/l)	1.56 ± 0.35	0.15 (0.10, 0.22)	< 0.001
NonHDLc (mmol/l)	3.08 ± 0.77	1.96 (1.71, 2.24)	< 0.001
NonHDLc/HDLc	2.10 ± 0.77	2.19 (1.94, 2.47)	< 0.001
ALT (U/L)	20.44 ± 11.04	1.04 (1.04, 1.05)	< 0.001
AST (U/L)	21.09 ± 6.54	1.04 (1.03, 1.05)	< 0.001

Data are expressed as the mean ± SD, median (interquartile range), or percentage

*BP* Blood pressure; *LDLc* Low-density lipoprotein cholesterol; *HDLc* High-density lipoprotein cholesterol; *ALT* Alanine aminotransferase; *AST* Aspartate aminotransferase

Hypertension was defined as blood pressure ≥ 140/90 mmHg or use of anti-hypertensive treatment

### Independent association between the baseline NonHDLc/HDLc ratio and the risk of NAFLD

Table 3 shows the results of the univariate and multivariate Cox proportional hazards models. In the univariate analysis, the HR for NAFLD significantly increased as the tertiles of the nonHDLc/HDLc ratio increased in both male and female. Compared with patients in the lowest tertile, female with nonHDLc/HDLc ratios in the highest tertile had a 1.66-fold increased risk of new-onset NAFLD (HR = 2.66; 95% CI, 1.13–6.24; *P* = 0.025), and male in this group had a 1.11-fold increased risk (HR = 2.11; 95% CI, 1.15–3.90; *P* = 0.016) after adjustment for age, BMI, DBP, SBP, fasting plasma glucose, TG,ALT, AST, and the development of cardiovascular disease or diabetes during follow-up.

**Table 3:** Risk association between baseline nonHDLc/HDLc ratio and NAFLD

Tertiles NonHDLc/HDLc	Unadjusted		Adjusted*	
	Female	Male	Female	Male
Tertile 1	1.0	1.0	1.0	1.0
Tertile 2	2.19 (1.08, 4.44), 0.031	1.85 (1.07, 3.19), 0.028	1.39 (0.60, 3.20), 0.441	1.27 (0.68, 2.38), 0.452
Tertile 3	7.14 (3.74, 13.62), < 0.001	4.60 (2.84, 7.44), < 0.001	2.66 (1.13, 6.24), 0.025	2.11 (1.15, 3.90), 0.016

Data are Hazard ratio (95% CI), *P* value

HDLc, high-density lipoprotein cholesterol

Adjusted* for age, body mass index, diastolic BP, systolic BP, fasting plasma glucose, TG,ALT, AST, development of cardiovascular disease or diabetes during follow-up

### The nonlinear relationship and saturation effect between the NonHDLc/HDLc ratio and the risk of NAFLD

**Fig. 1:**
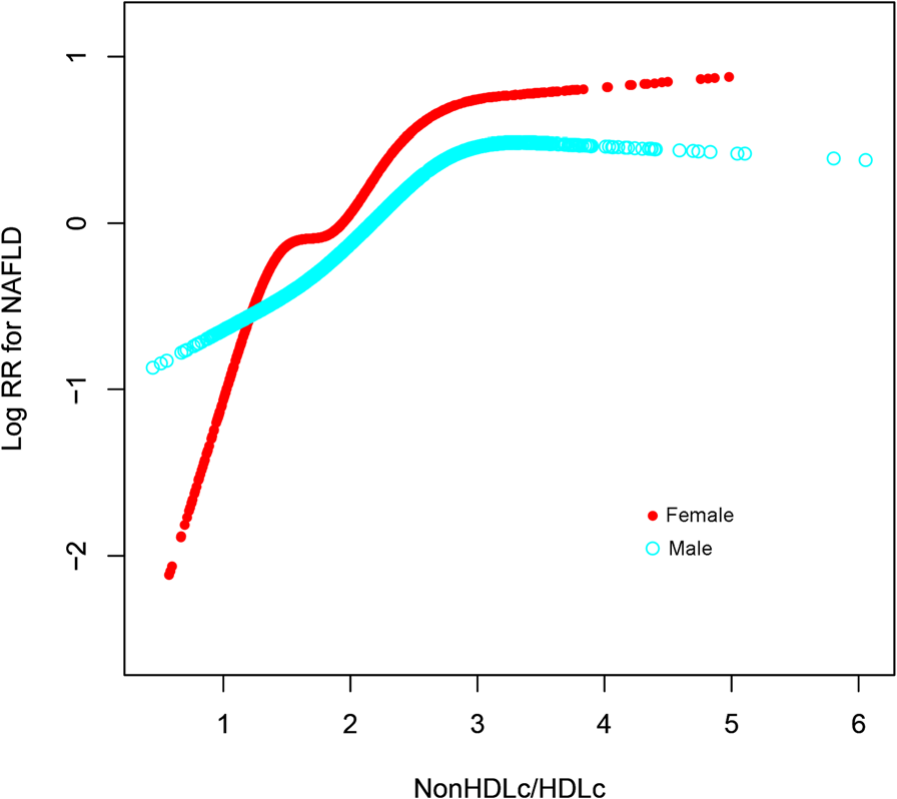
Baseline nonHDLc/HDLc ratio and the risk of NAFLD*. A nonlinear relationship between the nonHDLc/HDLc ratio and the risk of NAFLD was observed, and a saturated nonHDLc/HDLc ratio of 3.5 indicated a higher risk of NAFLD. The red line represents female, and the blue line represents male. *Adjusted for age, BMI, DBP, SBP, fasting plasma glucose, TG, ALT, AST, and development of cardiovascular disease or diabetes during follow-up

Figure 1 shows that in both female and male, a nonlinear relationship was observed between the nonHDLc/HDLc ratio and the risk of NAFLD after adjustment for potential confounders. The NAFLD risk increased with the nonHDLc/HDLc when nonHDLc/HDLc ratio was ≤3.5 for both genders. The HR for NAFLD was 2.15 (95% CI, 1.30–3.58; *P* = 0.003) for female and 1.62 (95% CI, 1.17–2.25; *P* = 0.004) for male with a nonHDLc/HDLc ratio ≤ 3.5, while it was 0.00 (95% CI, 0.00–45.96; *P* = 0.153) for female and 0.58 (95% CI, 0.18–1.86; *P* = 0.358) for male with a nonHDLc/HDLc ratio > 3.5. With the per-SD increase in the nonHDLc/HDLc ratio, the HR for NAFLD was 1.81 (95% CI, 1.22–2.68; *P* = 0.003) for female and 1.45 (95% CI, 1.13–1.87, *P* = 0.004) for male when nonHDLc/HDLc ratio was ≤3.5, while the HR was 0.00 (95% CI, 0.00–19.34; *P* = 0.153) for female and 0.65 (95% CI, 0.26–1.62; P = 0.358) for male when nonHDLc/HDLc ratio was > 3.5 (Table 4).

**Table 4:** The saturated effect of Baseline NonHDLc/HDLc ratio on the risk of NAFLD*

	Female		Male	
	Per-unit increase	Per-SD increase	Per-unit increase	Per-SD increase
NonHDLc/HDLc ratio ≤ 3.5	2.15 (1.30, 3.58), 0.003	1.81 (1.22, 2.68), 0.003	1.62 (1.17, 2.25), 0.004	1.45 (1.13, 1.87), 0.004
NonHDLc/HDLc ratio>3.5	0.00 (0.00, 45.96) 0.153	0.00 (0.00, 19.34), 0.153	0.58 (0.18, 1.86) 0.358	0.65 (0.26, 1.62), 0.358

Data are Hazard ratio (95% CI), *P* value

Adjusted* for age, body mass index, diastolic BP, systolic BP, fasting plasma glucose, TG,ALT, AST, development of cardiovascular disease or diabetes during follow-up

### The predictive value of the NonHDLc/HDLc ratio and NonHDLc value for NAFLD risk

**Fig. 2:**
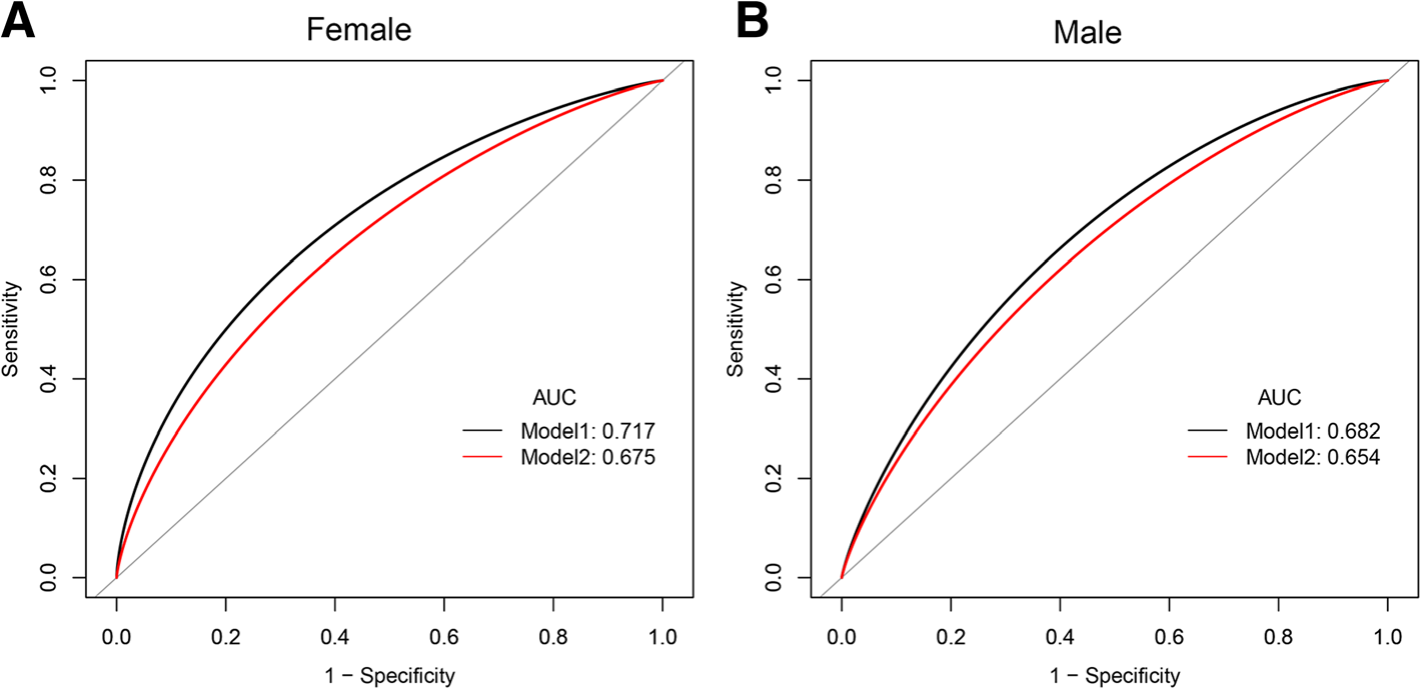
Receiver operating characteristic curves of nonHDLc/HDLc ratio and nonHDLc for NAFLD in female (**a**) and male (**b**). In female **a** the AUCs for the nonHDLc/HDLc ratio was 0.717 (95% CI, 0.656–0.776), while The AUCs for the nonHDLc value was 0.675 (95% CI, 0.613–0.736); In Male **b** the AUCs for the nonHDLc/HDLc ratio was 0.682 (95% CI, 0.644–0.722), while The AUCs for the nonHDLc value was 0.653 (95% CI, 0.609–0.691). The black line represents the nonHDLc/HDLc ratio, and the red line represents the nonHDLc value. AUC = area under the curve

To compare the predictive value of the nonHDLc/HDLc ratio and the nonHDLc value for NAFLD, we performed receiver operating characteristic (ROC) curve analyses (Fig. 2). The area under the curve (AUC) for the nonHDLc/HDLc ratio was 0.717 (95% CI, 0.656–0.776) in female and 0.682 (95% CI, 0.644–0.722) in male; while the AUC for the nonHDLc value was 0.675 (95% CI, 0.613–0.736) in female and 0.653 (95% CI, 0.609–0.691) in male. The AUCs for nonHDLc/HDLc ratio were significantly higher than those for nonHDLc value (*P* = 0.049 in female and *P* = 0.037 in male). The optimal cut-off value for the nonHDLc/HDLc ratio for identification of NAFLD in female was 2.4, with a sensitivity of 54.8% and a specificity of 79.9%, and the cut-off value in male was 2.3, with a sensitivity of 74.4% and a specificity of 57.9%.

## Discussion

Accumulating evidence suggested that the nonHDLc/HDLc ratio could predict multiple dyslipidemia-related disorders [13–15]. The pathogenesis of NAFLD has been confirmed to be associated with dyslipidemia [6, 7]. However, whether the ratio of nonHDLc to HDLc is an effective predictor of the incident NAFLD is still unknown. In this population-based perspective cohort study, we provided the first evidence demonstrating that the nonHDLc/HDLc ratio independently predicted new-onset NAFLD. Moreover, the nonHDLc/HDLc ratio showed a better predictive value than the nonHDLc in both female and male Chinese.

Convincing studies have demonstrated that dysregulation of cholesterol homeostasis is a pivotal metabolic factor in NAFLD pathogenesis [18]. Excess intracellular cholesterol can activate liver X receptors (LXRs), which could induce hepatic steatosis [19]. Meanwhile, free cholesterol loading, rather than free fatty acids or TG, can sensitize hepatocytes to inflammatory factors [20] and result in cytotoxicity-mediated transition of steatosis to NASH [21]. In addition, LXRs [18] can promote the secretion of very low-density lipoproteins (VLDLs). Increased VLDLs and the accompanying hypertriglyceridemia underlie the synthesis of small, dense LDLs with lower affinity for the LDL receptor. Oxidized LDLs could bind scavenger receptors and promote an inflammatory response. An overexpression of inflammatory cytokines could activate cholesterol synthesis and inhibit cholesterol elimination through bile acids, which together contribute to increases in LDLc and reductions in HDLc. Therefore, atherogenic dyslipidemia in the peripheral blood could represent cholesterol accumulation in hepatocytes and a higher risk of NAFLD. These could also partially explain the increased cardiovascular risk in patients with NAFLD.

NonHDLc includes all apoB-containing lipoproteins (VLDLs, LDLc, intermediate density lipoprotein, chylomicrons and lipoprotein A) and was demonstrated as a more powerful marker for CVD and NAFLD than than single lipoprotein [22]. In contrast to the nonHDLc value, the nonHDLc/HDLc ratio was suggested to better represent the balance between proatherogenic and antiatherogenic lipoproteins and indicate a more comprehensive lipid dysregulation. Studies have proven the NonHDLc/HDLc ratio to be a better predictor for CVD than NonHDLc [23], as well as a better predictor for insulin resistance and MS than the apoB/apoA1 ratio (difference might be attributed to dysfunctional HDL levels). [14]

In this study, we demonstrated that the nonHDLc/HDLc ratio is an independent predictor of NAFLD and a stronger predictor than nonHDLc for the first time. Our study is consistent with previous trials and contributes to interpretation of the cause-effect relationship between dyslipidemia and NAFLD.

Our study found that the female has a more significant association with the risk of NAFLD than the male. Our result is consistent with the studies of Fernandes et al. [24] and Haentjens et al. [25], who showed that female have a higher risk of developing NAFLD than male. Astrid et al. [26] suggested that enhanced liver damage found in female mice may result from alterations of the adiponectin–AMPK–PAI-1 signalling cascade in the liver. Further research should be conducted to determine the differences in the pathophysiological mechanisms between male and female with NAFLD. We also found a saturated effect of the nonHDLc/HDLc ratio on the risk of NAFLD, which may be due to saturation of receptors and enzymes in the liver. Further studies are needed to establish the exact molecular association between cholesterol alterations and NAFLD.

The results of this study have several potential clinical implications. The nonHDLc/HDLc ratio may serve as a more effective biomarker for predicting and treating NAFLD than traditional blood lipid parameters. The optimal cutoff values of the nonHDLc/HDLc ratio for identifying individuals with NAFLD (in women was 2.4 and in men was 2.3) may play roles in screening high-risk groups with NAFLD in a Chinese population. In addition, the NonHDLc value is readily available from a routine fasting lipid profile regardless of the TG level, thus reducing the need for additional and more expensive apoB diagnostic tests.

Several limitations should be noted in our study. First, NAFLD was diagnosed by ultrasonography rather than liver histopathology, which may lead to an inaccurate diagnosis. Nevertheless, liver ultrasonography has been confirmed as an accurate and reliable tool for detecting fatty liver. Due to the relatively low cost and lack of radiation exposure, ultrasound is the choice imaging modality for screening for fatty liver in clinical settings and population studies. Second, the interobserver variation might occur for we did not assess the reproducibility of the interpretations of ultrasonographers. However, because all ultrasonographers were blinded to the study information, misclassification would be random. Third, although we adjusted for multiple potential confounding variables, residual and unmeasured confounding might not be fully addressed. Moreover, since all subjects in our study were Asians, results of this study might not directly be applicable to other ethnicities. More prospective multicenter and larger-scale studies are needed to clarify our conclusions in the future.

## Conclusion

In conclusion, this perspective study performed in the Chinese population revealed that the increase in nonHDLc/HDLc ratio independent predicts the new-onset NAFLD, and performs better than the single nonHDLc value. The stronger predictor would be expected to better guide early identification of individuals at risk of NAFLD and help contribute to prevent the development and progression of this disease.

## Abbreviations

ALT: Alanine aminotransferase
ApoB: Apolipoprotein
AST: Aspartate aminotransferase
AUC: Calculated the areas under the curve
BMI: Body mass index
CHD: Coronary heart disease
CVD: Cardiovascular diseases
DBP: Diastolic blood pressure
HDLc: High-density lipoprotein cholesterol
HR: Hazard ratio
LDLc: Low-density lipoprotein cholesterol
NAFLD: Non-alcoholic fatty liver disease
NASH: Non-alcoholic steatohepatitis
nonHDLc/HDLc ratio: The nonHDLc to HDLc ratio
ROC: Receiver operating characteristic
SBP: Systolic blood pressure
TC: Total cholesterol
TG: Triglycerides

## References

[1] Lazo M, Hernaez R, Eberhardt MS, Bonekamp S, Kamel I, Guallar E, Koteish A, Brancati FL, Clark JM (2013). Prevalence of nonalcoholic fatty liver disease in the United States: the third National Health and nutrition examination survey, 1988-1994. Am J Epidemiol.

[2] Rinella ME (2015). Nonalcoholic fatty liver disease. JAMA.

[3] Fan JG, Kim SU, Wong VW (2017). New trends on obesity and NAFLD in Asia. J Hepatol.

[4] Farrell GC, Wong VW, Chitturi S (2013). NAFLD in Asia--as common and important as in the west. Nat Rev Gastroenterol Hepatol.

[5] Chalasani N, Younossi Z, Lavine JE, Diehl AM, Brunt EM, Cusi K, Charlton M, Sanyal AJ (2012). The diagnosis and management of non-alcoholic fatty liver disease: practice guideline by the American Gastroenterological Association, American Association for the Study of Liver Diseases, and American College of Gastroenterology. GASTROENTEROLOGY.

[6] Cohen DE, Fisher EA (2013). Lipoprotein metabolism, dyslipidemia, and nonalcoholic fatty liver disease. Semin Liver Dis.

[7] Speliotes EK, Massaro JM, Hoffmann U, Vasan RS, Meigs JB, Sahani DV, Hirschhorn JN, O'Donnell CJ, Fox CS (2010). Fatty liver is associated with dyslipidemia and dysglycemia independent of visceral fat: the Framingham heart study. HEPATOLOGY.

[8] DeFilippis AP, Blaha MJ, Martin SS, Reed RM, Jones SR, Nasir K, Blumenthal RS, Budoff MJ (2013). Nonalcoholic fatty liver disease and serum lipoproteins: the multi-ethnic study of Atherosclerosis. ATHEROSCLEROSIS.

[9] Bedogni G, Miglioli L, Masutti F, Castiglione A, Croce LS, Tiribelli C, Bellentani S (2007). Incidence and natural course of fatty liver in the general population: the Dionysos study. HEPATOLOGY.

[10] Hamaguchi M, Kojima T, Takeda N, Nakagawa T, Taniguchi H, Fujii K, Omatsu T, Nakajima T, Sarui H, Shimazaki M (2005). The metabolic syndrome as a predictor of nonalcoholic fatty liver disease. Ann Intern Med.

[11] Omagari K, Kadokawa Y, Masuda J, Egawa I, Sawa T, Hazama H, Ohba K, Isomoto H, Mizuta Y, Hayashida K (2002). Fatty liver in non-alcoholic non-overweight Japanese adults: incidence and clinical characteristics. J Gastroenterol Hepatol.

[12] Zelber-Sagi S, Salomone F, Yeshua H, Lotan R, Webb M, Halpern Z, Santo E, Oren R, Shibolet O (2014). Non-high-density lipoprotein cholesterol independently predicts new onset of non-alcoholic fatty liver disease. Liver Int.

[13] Zhu L, Lu Z, Zhu L, Ouyang X, Yang Y, He W, Feng Y, Yi F, Song Y (2015). Lipoprotein ratios are better than conventional lipid parameters in predicting coronary heart disease in Chinese Han people. Kardiol Pol.

[14] Kim SW, Jee JH, Kim HJ, Jin S, Suh S, Bae JC, Kim SW, Chung JH, Min Y, Lee M (2013). Non-HDL-cholesterol/HDL-cholesterol is a better predictor of metabolic syndrome and insulin resistance than apolipoprotein B/apolipoprotein A1. Int J Cardiol.

[15] Eliasson B, Cederholm J, Eeg-Olofsson K, Svensson AM, Zethelius B, Gudbjornsdottir S (2011). Clinical usefulness of different lipid measures for prediction of coronary heart disease in type 2 diabetes: a report from the Swedish National Diabetes Register. Diabetes Care.

[16] Appropriate body-mass index for Asian populations and its implications for policy and intervention strategies. LANCET. 2004;363(9403):157–63. 10.1016/S0140-6736(03)15268-3 14726171

[17] Hernaez R, Lazo M, Bonekamp S, Kamel I, Brancati FL, Guallar E, Clark JM (2011). Diagnostic accuracy and reliability of ultrasonography for the detection of fatty liver: a meta-analysis. HEPATOLOGY.

[18] Fon TK, Rozman D (2011). Nonalcoholic fatty liver disease: focus on lipoprotein and lipid deregulation. J Lipids.

[19] Ducheix S, Montagner A, Theodorou V, Ferrier L, Guillou H (2013). The liver X receptor: a master regulator of the gut–liver axis and a target for non alcoholic fatty liver disease. Biochem Pharmacol.

[20] Mari M, Caballero F, Colell A, Morales A, Caballeria J, Fernandez A, Enrich C, Fernandez-Checa JC, Garcia-Ruiz C (2006). Mitochondrial free cholesterol loading sensitizes to TNF- and Fas-mediated steatohepatitis. Cell Metab.

[21] Van Rooyen DM, Larter CZ, Haigh WG, Yeh MM, Ioannou G, Kuver R, Lee SP, Teoh NC, Farrell GC (2011). Hepatic free cholesterol accumulates in obese, diabetic mice and causes nonalcoholic steatohepatitis. Gastroenterology.

[22] Hermans MP, Sacks FM, Ahn SA, Rousseau MF (2011). Non-HDL-cholesterol as valid surrogate to apolipoprotein B100 measurement in diabetes: discriminant ratio and unbiased equivalence. Cardiovasc Diabetol.

[23] Taskinen MR, Barter PJ, Ehnholm C, Sullivan DR, Mann K, Simes J, Best JD, Hamwood S, Keech AC (2010). Ability of traditional lipid ratios and apolipoprotein ratios to predict cardiovascular risk in people with type 2 diabetes. DIABETOLOGIA.

[24] Fernandes MT, Ferraro AA, de Azevedo RA, Fagundes NU (2010). Metabolic differences between male and female adolescents with non-alcoholic fatty liver disease, as detected by ultrasound. Acta Paediatr.

[25] Haentjens P, Massaad D, Reynaert H, Peeters E, Van Meerhaeghe A, Vinken S, Poppe K, Velkeniers B (2009). Identifying non-alcoholic fatty liver disease among asymptomatic overweight and obese individuals by clinical and biochemical characteristics. Acta Clin Belg.

[26] Spruss A, Henkel J, Kanuri G, Blank D, Puschel GP, Bischoff SC, Bergheim I (2012). Female mice are more susceptible to nonalcoholic fatty liver disease: sex-specific regulation of the hepatic AMP-activated protein kinase-plasminogen activator inhibitor 1 cascade, but not the hepatic endotoxin response. Mol Med.

